# Maternal Mortality Underreporting in Brazil Due to Coding Errors (2010–2021)

**DOI:** 10.1097/AOG.0000000000006274

**Published:** 2026-04-02

**Authors:** Ana Maria Pearce de Arêa Leão Pinheiro, Rafael Sant'Ana Herzog, Regina Amélia Lopes Pessoa de Aguiar, Agatha Sacramento Rodrigues, Rossana Pulcineli Vieira Francisco

**Affiliations:** Brazilian Obstetric Observatory (OOBr), Divisão de Obstetrícia, Departamento de Obstetrícia e Ginecologia, Faculdade de Medicina, Universidade de São Paulo, São Paulo, Divisão de Obstetrícia, Departamento de Obstetrícia e Ginecologia, Universidade Federal do Piauí, Teresina, Departamento de Estatística, Universidade Federal do Espírito Santo, Vitória, and Departamanto de Ginecologia e Obstetrícia, Universidade Federal de Minas Gerais, Belo Horizonte, Brazil.

## Abstract

Accurate maternal death tracking is a global priority to guide resource allocation for reduction of preventable maternal mortality. This retrospective observational study evaluated the accuracy of the Brazilian maternal death coding system and compared the official maternal mortality ratio (MMR) with a recalculated MMR including any reclassified maternal deaths. Mortality data from 2010 to 2021 were analyzed, including officially reported maternal deaths and deaths of women aged 10–49 years with pregnancy–puerperal status indicated on their death certificate but not coded as maternal. Brazil recorded 21,670 official and 3,480 uncoded maternal deaths during the study period. Inclusion of reclassified maternal deaths increased the median MMR from 58.2 to 67.5 per 100,000 live births. Uncoded maternal deaths compared with coded deaths were more likely among those with lower education, at younger and older maternal ages, with indirect causes of death, and with fewer death investigations. Improved maternal death coding is essential to reduce maternal mortality underreporting.

## Abstract 2

When deaths not classified as maternal due to coding errors were reclassified as maternal deaths, the maternal mortality ratio in Brazil increased.

Maternal deaths include any deaths occurring during pregnancy or within 42 days of pregnancy termination, excluding accidental or incidental causes.^1^ Maternal deaths are an important global metric to inform maternal mortality ratios (MMRs) and direct resources to reduce preventable maternal mortality.^2^ The World Health Organization has called for improved measurement systems to ensure the accuracy of maternal death reporting.^2^

In Brazil, maternal deaths are registered in accordance with International Classification of Diseases, Tenth Revision (ICD-10) chapter XV coding. Use of pregnancy–puerperal checkboxes on death certificates (ie, identifying death occurred during pregnancy, childbirth, or the puerperium) should result in automatic ICD-10 coding as a maternal death.^3^ However, there is a risk for coding errors, and studies have not addressed the potential for underreporting of maternal deaths in Brazil. Therefore, we aimed to evaluate the accuracy of the Brazilian maternal death coding system. Additionally, we aimed to compare the officially reported MMR with the MMR including any reclassified maternal deaths.

## METHODS

This was a retrospective observational study using publicly available data from the Brazilian Mortality Information System^4^ on all deaths of women aged 10–49 years from 2010 to 2021. *Official maternal deaths* were defined as those coded under ICD-10 chapter XV (A34, B20–B24, E23.0, M83.0, F53, and D39.2) by the Ministry of Health, according to national guidelines.^3^ Uncoded maternal deaths were those with the pregnancy–puerperal checkbox marked on the death certificate but not coded as a maternal death under ICD-10 chapter XV, excluding external causes.

Baseline characteristics were compared between official and uncoded maternal deaths, including age, education, marital status, place of death, type of maternal death, investigation status, and access to care. Uncoded deaths were recoded using ICD-10 chapter XV codes (Appendix 1, available online at http://links.lww.com/AOG/E611)^1^ and classified as direct, indirect, or attributed to unspecified obstetric causes (Appendix 2, available online at http://links.lww.com/AOG/E611).^5^

After reclassification of maternal deaths, the MMRs were recalculated by federal unit. Median differences between the officially reported MMR and the MMR with reclassified maternal deaths were assessed using the Wilcoxon signed-rank test. Associations were assessed by estimating odds ratios with 95% CIs using unadjusted and adjusted logistic regression models, with reported maternal deaths as the reference. Adjusted models included the covariate of interest and remaining variables as potential confounders. Missing data in the main exposure were excluded; other missing values were retained as a separate category. Analyses were conducted in R statistical software^6^ with a 5% significance level.

We followed the STROBE (Strengthening the Reporting of Observational Studies in Epidemiology) reporting guidelines. Ethics committee approval was not required because the study used de-identified, publicly available data, in accordance with National Health Council Resolution 510/2016.

## RESULTS

**Fig. 1.**
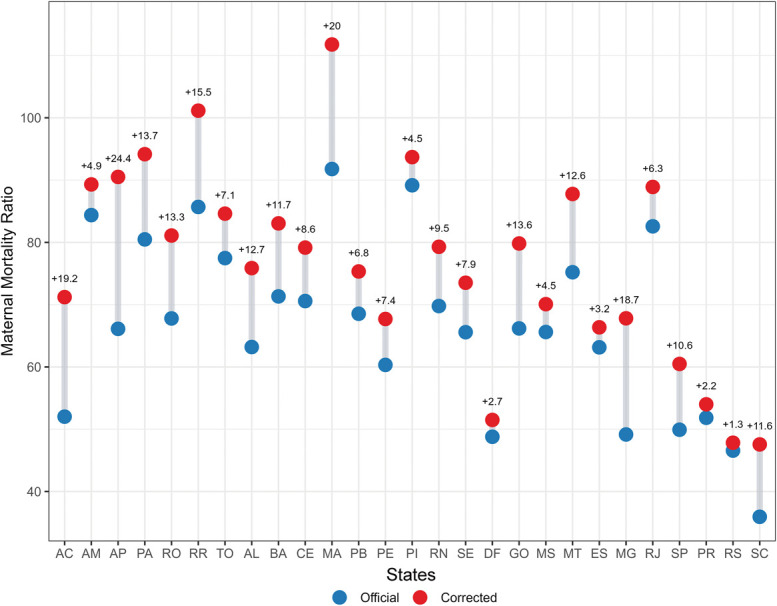
Difference between the official and corrected maternal mortality ratios by state, 2010–2021. AC, Acre; AM, Amazonas; AP, Amapá; PA, Pará; RO, Rondônia; RR, Roraima; TO, Tocantins; AL, Alagoas; BA, Bahia; CE, Ceará; MA, Maranhão; PB, Paraíba; PE, Pernambuco; PI, Piauí; RN, Rio Grande do Norte; SE, Sergipe; DF, Federal District; GO, Goiás; MS, Mato Grosso do Sul; MT, Mato Grosso; ES, Espírito Santo; MG, Minas Gerais; RJ, Rio de Janeiro; SP, São Paulo; PR, Paraná; RS, Rio Grande do Sul; SC, Santa Catarina.

From 2010 to 2021, 21,670 maternal deaths were officially reported in Brazil, with 3,480 additional deaths initially uncoded as maternal. Inclusion of uncoded cases increased the median national MMR from 58.2 to 67.5 per 100,000 live births, with the largest relative increases in Amapá, Maranhão, and Acre (Fig. 1).

*Pinheiro. Maternal Mortality Underreporting in Brazil. Obstet Gynecol 2026*.

The most frequent underlying causes of death among uncoded deaths were ill-defined causes (Appendix 3, available online at http://links.lww.com/AOG/E611). After recoding, leading classifications were circulatory (O99.4) and respiratory (O99.5) diseases complicating pregnancy (Appendix 4, available online at http://links.lww.com/AOG/E611).

Univariate analyses showed no differences in race or autopsy performance by coded compared with uncoded maternal deaths, but differences were observed for other characteristics (Table 1). In multivariable models, uncoded deaths were more likely at younger and older maternal ages (10–14 years and 45–49 years), among women with lower maximum education, outside hospital settings, and as a result of indirect or unspecified obstetric causes. Uncoded maternal deaths were also less likely to have undergone committee investigation and were more often without autopsy (Table 1).

**Table 1.**
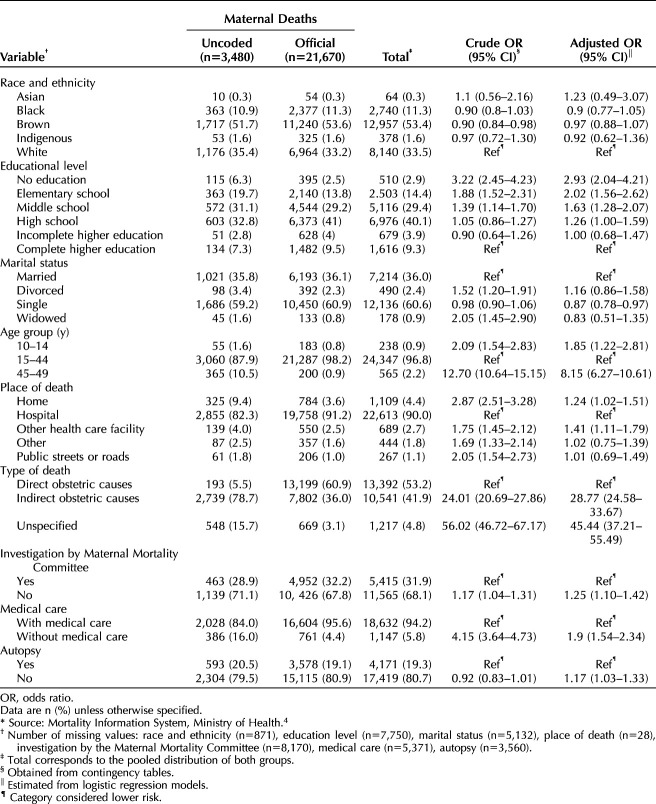
Baseline Characteristics of Uncoded and Official Maternal Deaths in Brazil, 2010–2021*

## DISCUSSION

The current ICD-10–based classification system substantially underestimated maternal deaths in Brazil. Reclassifying deaths identified through the death certificate pregnancy checkbox increased the national MMR by approximately 16%, consistent with international evidence of underreporting due to coding errors.^7,8^

The predominance of indirect causes among uncoded maternal deaths underscores diagnostic and classification challenges, particularly for cardiovascular, respiratory, and infection conditions exacerbated by pregnancy.^9,10^ The low proportion of investigations among uncoded maternal deaths may indicate that deaths were not adequately reviewed. Uncoded deaths disproportionately affected socially vulnerable women and regions with weaker surveillance systems, reinforcing the role of social inequities in maternal mortality measurement.^10^

A limitation of this study is that maternal deaths could not be verified through source records. The death certificate pregnancy checkbox increased the number of maternal deaths recognized, but the checkbox may generate false-positive results. Other studies have noted limitations in death certificate checkbox accuracy but emphasized its utility in identifying missed cases.^11^

Accurate maternal death classification is essential to guide public health policies and monitor progress toward maternal mortality reduction targets.^2^ Our findings highlight concerns with the current process in Brazil. Improved maternal death reporting may be achieved through targeted training of coders, implementation of automated systems, or other mechanisms.

## Supplementary Material
